# Anoctamin 6 is localized in the primary cilium of renal tubular cells and is involved in apoptosis-dependent cyst lumen formation

**DOI:** 10.1038/cddis.2015.273

**Published:** 2015-10-08

**Authors:** V Forschbach, M Goppelt-Struebe, K Kunzelmann, R Schreiber, R Piedagnel, A Kraus, K-U Eckardt, B Buchholz

**Affiliations:** 1 Department of Nephrology and Hypertension, Friedrich-Alexander-University Erlangen-Nuernberg, 91054 Erlangen, Germany; 2 Department of Physiology, University of Regensburg, 93053 Regensburg, Germany; 3 Sorbonne Universités, UPMC Univ Paris 06, UMR_S 1155, F-75005 Paris, France; 4 INSERM, UMR_S 1155, F-75005 Paris, France

## Abstract

Primary cilia are antenna-like structures projected from the apical surface of various mammalian cells including renal tubular cells. Functional or structural defects of the cilium lead to systemic disorders comprising polycystic kidneys as a key feature. Here we show that anoctamin 6 (ANO6), a member of the anoctamin chloride channel family, is localized in the primary cilium of renal epithelial cells *in vitro* and *in vivo*. ANO6 was not essential for cilia formation and had no effect on *in vitro* cyst expansion. However, knockdown of ANO6 impaired cyst lumen formation of MDCK cells in three-dimensional culture. In the absence of ANO6, apoptosis was reduced and epithelial cells were incompletely removed from the center of cell aggregates, which form in the early phase of cystogenesis. In line with these data, we show that ANO6 is highly expressed in apoptotic cyst epithelial cells of human polycystic kidneys. These data identify ANO6 as a cilium-associated protein and suggest its functional relevance in cyst formation.

Primary cilia are non-motile protrusions of the apical membrane of various cell types.^1^ The ciliary membrane contains receptors and ion channels that link mechanical or chemical stimuli including fluid flow, sonic hedgehog and growth factors to intracellular signaling cascades regulating cell differentiation, migration and growth.^2^ Mutations in genes encoding for proteins that are necessary either for the function or the structure of the primary cilium lead to ciliopathies, systemic disorders that are typically characterized by the development of polycystic kidneys.^3^

Anoctamins (ANO1-ANO10, TMEM16A-K) form a family of 10 proteins that are supposed to act as Ca^2+^-activated chloride channels with no homology to other known ion channels.^4, 5^ In contrast to the other paralogues, the function of ANO1 and ANO2 as Ca^2+^-activated chloride channels has been confirmed *in vivo* and *in vitro*.^6, 7, 8^ Besides its ability to conduct ions ANO6 has also been shown to act as a phospholipid scramblase.^9, 10^ ANO3, 4, 7, 9 may also act as Ca^2+^-dependent phospholipid scramblases.^11^ However, data about their functional roles are very limited so far. ANO6 is the most widely expressed paralogue.^12^ Mutations in ANO6 cause the Scott syndrome, which is characterized by a defect in Ca^2+^-dependent phospholipid scrambling of plasma membrane phospholipids.^10^ In addition, ANO6 is involved in bone mineralization, cell volume regulation, cell proliferation and apoptosis.^12^ Despite the broad expression and function of ANO6, there is only sparse data about the subcellular localization of ANO6. Recently, we have shown that ANO6 is expressed in cyst-forming epithelial cells together with ANO1, which is widely expressed in epithelial cells.^13^ Knockdown of ANO1 but not ANO6 significantly reduced secretion-dependent cyst growth pointing towards distinct functions of ANO1 and ANO6 in the cyst epithelium.^13^

Lumen formation of different epithelial cell types represents a fundamental step for the proper development of several organs including lungs, pancreas, intestine and kidneys.^14^ It comprises complex cell–cell and cell–matrix recognition, establishment of apical–basal polarity as well as cavitation, which depends on apoptosis of cells situated within the lumen.^14^ The mechanisms involved in apoptosis-dependent cavitation are incompletely understood. Interestingly, in polycystic kidneys, a prime example of misled lumen formation leading to cystogenesis and subsequent cyst growth, cyst epithelial cells show increased levels of apoptosis.^15^

This study was conducted to determine the subcellular localization of ANO6 in renal tubular cells and a possible role of this protein in cyst formation.

## Results

### Anoctamin 6 is localized in the primary cilium of renal tubular cells

**Figure 1:**
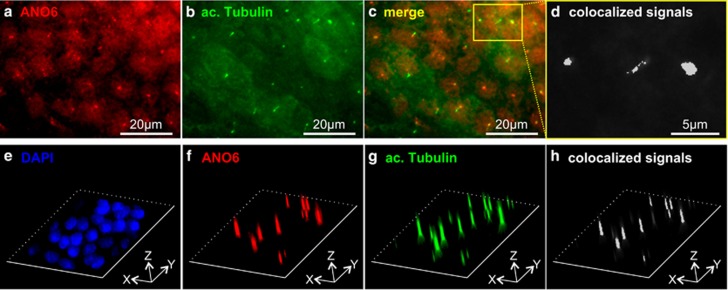
Anoctamin 6 is localized in the plasma membrane and the primary cilium of MDCK cells. (**a**) MDCK cells grown on permeable supports were stained for ANO6 providing round distinct signals; (**b**) cells in **a** were stained for the ciliary marker acetylated tubulin; (**c**) merged photo of **a** and **b** are shown; (**d**) calculated colocalization in white of the magnified section marked in **c** are shown as described in the Materials and Methods section; (**e**–**h**) three-dimensional illustration of MDCK cells stained for nuclei (DAPI, **e**), ANO6 (**f**) and primary cilia (acetylated tubulin, **g**); (**h**) calculated colocalization of ANO6 and acetylated tubulin highlighted in white

**Figure 2:**
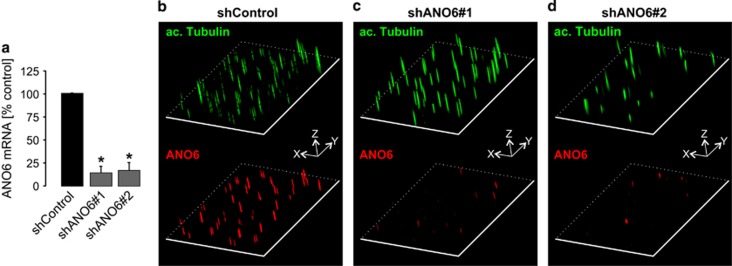
MDCK cells stably deficient for ANO6 show reduced signal of ANO6 within the cilium. (**a**) Downregulation of ANO6 mRNA in two MDCK cell clones (clone 3 (cl.3) and clone 7 (cl.7)) stably transfected with two distinct shRNAs directed against ANO6 (shANO6#1 and shANO6#2) compared with MDCK cells stably transfected with scrambled shRNA (shControl) serving as control, *P<0.05; (**b**–**d**) three-dimensional illustration of MDCK cells described in **a** stained for acetylated tubulin (upper panel) and ANO6 (lower panel)

**Figure 3:**
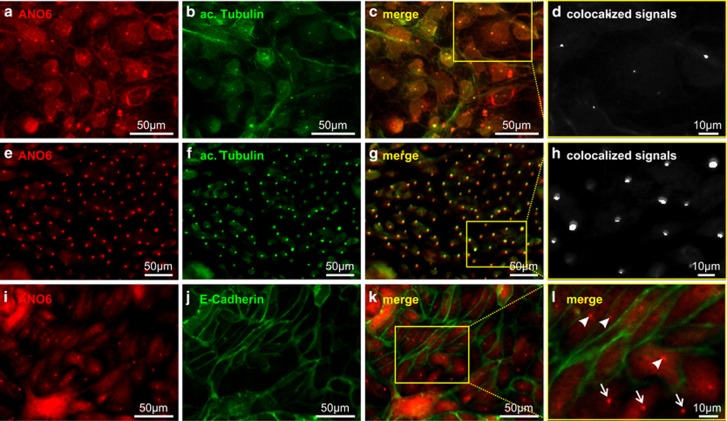
ANO6 localizes in the primary cilium of a human collecting duct cell line (HCD) and human primary tubule cells. HCD cells were grown on permeable supports and stained for ANO6 (**a**) and acetylated tubulin (**b**); (**c**) merged photo of **a** and **b** is shown; (**d**) calculated colocalization highlighted in white of the magnified section marked in **c** is shown; primary human renal tubule cells were stained for ANO6 (**e**) and acetylated tubulin (**f**); (**g**) merged photo of **e** and **f** is shown; (**h**) calculated colocalization highlighted in white of the magnified section marked in **g** is shown; primary human renal tubule cells were stained for ANO6 (**i**) and the distal tubular cell marker E-cadherin (**j**); (**k**) merged photo of **i** and **j** is shown; (**l**) magnified section marked in **k** is shown. Arrowheads mark ANO6-positive cells stained positive for E-cadherin. Arrows mark ANO6-positive cells stained negative for E-cadherin

ANO6 localization was analyzed in canine and human renal tubular cells. For this purpose, we developed and characterized three different antibodies as described in Materials and Methods. First, we analyzed the subcellular localization of endogenous ANO6 in polarized Madin-Darby Canine Kidney (MDCK) cells, which originate from collecting duct cells. We found distinct signals in dense, polarized cells grown on permeable supports that colocalized with acetylated tubulin, a marker for the primary cilium (Figures 1a–h). Comparable results were obtained with each of the three antibodies (Supplementary Figures 1A–F). In addition, ANO6 also seemed to be expressed in the plasma membrane (Supplementary Figures 1G–I). To confirm the specificity of these findings, we next generated MDCK cell clones stably expressing one of two shRNAs directed against ANO6 (shANO6#1 and shANO6#2) or control shRNA (shCtrl), respectively. Both, shANO6#1 and shANO6#2 provided a significant reduction of ANO6 expression of more than 80% (Figure 2a). Of note, both cell clones still formed cilia (Figures 2c and d) but showed a marked reduction of ciliary ANO6 signal (Figures 2b–d). This finding confirmed ciliary localization of ANO6, but indicated that ANO6 is not essential for cilium formation. Next, we tested for the localization in human tubular cells to exclude cell line- or species-specific localization of ANO6. In Human Collecting Duct (HCD) cells,^16^ we found identical membranous and ciliary staining patterns as in MDCK cells (Figures 3a–d). In addition, we also analyzed primary human tubular cells isolated from nephrectomized kidneys comprising proximal, distal and collecting duct cells^17^ (Figures 3e–h and Supplementary Figure 2). To distinguish cells originating from different tubular segments, we took advantage of the fact that human proximal epithelial cells uniquely express N-cadherin instead of E-cadherin as major cell–cell adhesion molecule.^17^ Ciliary localization of ANO6 was found irrespective of the tubular origin (Figures 3i–l) indicating ciliary expression of ANO6 in different tubular segments.

### ANO6 is involved in apoptosis-dependent lumen formation of MDCK cysts

**Figure 4:**
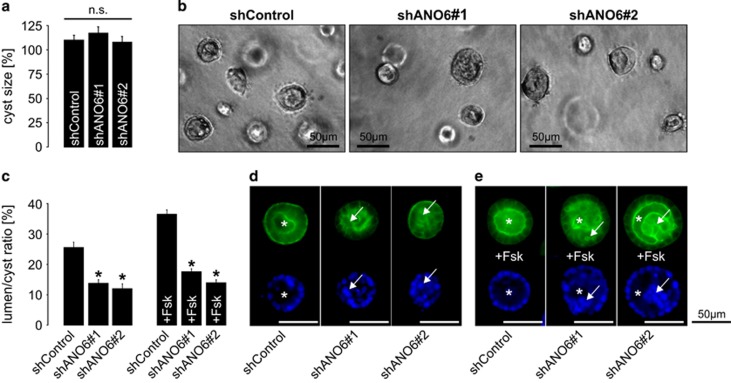
MDCK cells deficient for ANO6 show disturbed lumen formation within a collagen matrix. Non-transfected MDCK cells as well as stable control-transfected MDCK cells (shControl) and MDCK cells stably deficient for ANO6 (shANO6#1 and shANO6#2) were grown within a collagen I matrix in the presence and absence of 10 *μ*M forskolin (Fsk) to form cysts for 5 days. (**a**) Cyst sizes of control-transfected MDCK cells and ANO6-deficient cells±S.E.M. in the presence of 10 *μ*M forskolin relative to non-tranfected cells (set 100%) from three individual experiments comprising the analysis of ~75–125 cysts per condition; (**b**) representative cysts within the collagen matrix at day 5 are shown; (**c**) ratio of luminal area and cyst area±S.E.M. in the presence and absence of 10 *μ*M forskolin (Fsk) was determined in cysts described in **a** from three individual experiments comprising the analysis of ~75–125 cysts per condition, *P<0.05; (**d**) representative cysts in the absence and (**e**) presence of 10 *μ*M forskolin (Fsk) stained for F-actin (upper panel) and DAPI (lower panel) are shown. * represents proper cyst lumen, arrows point at cells situated within the cyst lumen

To test for a functional role of ANO6 in cyst formation, we used the MDCK cyst model.^18^ Both, control-transfected MDCK cells as well as MDCK cells stably deficient for ANO6 formed cysts within a collagen matrix and showed comparable cyst sizes in the presence of forskolin (Figures 4a and b). This suggests that ANO6 is not essential for fluid secretion into the cyst lumen, the main mechanism of cyst expansion, although forskolin led to a significant translocation of ANO6 from the cytosol towards the apical membrane (Supplementary Figures 3A and B). This was further confirmed by Ussing chamber experiments where MDCK cells stably deficient for ANO6 showed unaffected transepithelial chloride secretion upon treatment with either ATP or IBMX/forskolin compared with control-transfected cells (Supplementary Figures 3C–F). However, in cysts derived from MDCK cells lacking ANO6, lumen formation was incomplete. This was caused by an increased number of cells situated within the cyst lumen, and reflected by a significant reduction of the lumen-to-cyst ratio (Figures 4c–e). In accordance with our previous findings,^13^ the lumen-to-cyst ratio was augmented in the presence of forskolin in both ANO6-competent and ANO6-deficient cells (Figures 4c–e), again reflecting preserved lumen expansion owing to transepithelial chloride secretion.

Next, we were interested to determine the mechanisms underlying the increased number of cells within the cyst lumen in ANO6-deficient cells. We tested whether intracystic cell accumulation was owing to increased cell proliferation of ANO6-deficient cells. However, cell proliferation in ANO6-deficient MDCK cells was reduced (Supplementary Figure 3G), which is in line with a previous report, where ANO6 has been shown to be involved in cell proliferation of myoblasts.^19^

**Figure 5:**
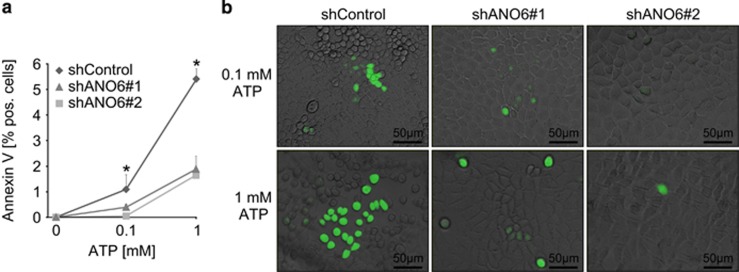
ANO6 is involved in calcium-dependent phospholipid scrambling of MDCK cells. (**a**) Stably control-transfected MDCK cells (shControl) and MDCK cells stably deficient for ANO6 (shANO6#1 and shANO6#2) were grown on cell culture dishes. After incubation with either 0, 0.1 or 1 mM ATP, FITC-labeled annexin V was added and the fraction of annexin V-positive cells was analyzed, *P<0.05. (**b**) representative photos of the cells described in **a** (green: annexin V-FITC) are shown

**Figure 6:**
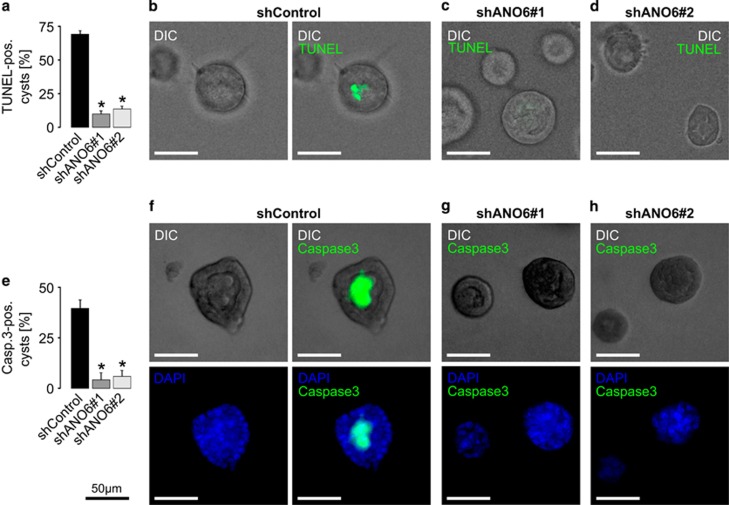
ANO6 is involved in apoptosis-dependent lumen formation of MDCK cysts. Stable control-transfected MDCK cells (shControl) and MDCK cells stably deficient for ANO6 (shANO6#1 and shANO6#2) were grown within a collagen I matrix to form cysts for 3 days where lumen formation and cavitation is still taking place in control cysts. Then cysts were photographed by the use of digital interference contrast (DIC) and stained for apoptosis by the use of TUNEL (**a**–**d**) and antibodies directed against cleaved caspase 3 (**e**–**h**). (**a**) Quantification of TUNEL-positive cysts from three individual experiments comprising 65–85 cysts per condition; (**b**) left: representative ANO6-competent cyst, right: overlay with TUNEL staining; (**c**, **d**) representative ANO6-deficient cysts with overlay of TUNEL staining; (**e**) quantification of cleaved caspase 3-positive cysts from three individual experiments comprising ~50 cysts per condition; (**f**) upper panel, left: representative ANO6-competent cyst, right: overlay with staining for cleaved caspase 3; lower panel, left: control cyst stained with DAPI, right: shows merge of DAPI stain and cleaved caspase 3; (**g**, **h**) upper panel: overlay of representative ANO6-deficient cysts and staining for cleaved caspase 3; lower panel shows merge of DAPI stain and cleaved caspase 3, *P<0.05

As ANO6 has been reported to be pro-apoptotic in lymphocytes, macrophages and platelets,^20, 21, 22^ and *in vitro* lumen formation is based on apoptosis-dependent cavitation,^14, 23^ we next examined apoptosis in ANO6-deficient cells. During apoptosis, ANO6 is necessary for calcium-dependent phospholipid scrambling resulting in an increased presentation of negatively charged phosphatidylserines at the outer membrane leaflet.^20^ This can be visualized by binding of annexin V protein to negatively charged phosphatidylserines.^18^ Indeed and in accordance with previous findings, lack of ANO6 significantly reduced calcium-dependent binding of annexin V in MDCK cells (Figure 5). We then compared the expression of apoptotic markers during the early stages of MDCK lumen formation of ANO6-competent and ANO6-deficient cells. Strikingly, luminal cells in ANO6-deficient MDCK cysts were largely negative for TUNEL staining as well as for activated caspase 3, whereas luminal cells of ANO6-competent cysts were highly positive for these apoptotic markers (Figure 6). These data depict ANO6 as a pro-apoptotic protein that is involved in MDCK cyst lumen formation.

### ANO6 is expressed in cyst-lining epithelial cells of polycystic kidneys

**Figure 7:**
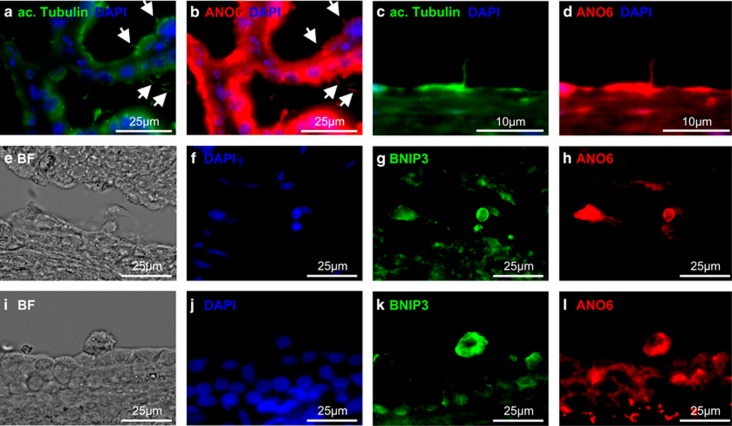
ANO6 is localized in the primary cilia of renal tubule cells and strongly expressed in apoptotic cyst epithelial cells *in vivo*. Sections of human polycystic kidneys were stained for acetylated tubulin (**a** and **c**) and ANO6 (**b** and **d**); (**e** and **i**) brightfield view of sections of human polycystic kidneys are shown, further stained with DAPI (**f** and **j**), the apoptotic marker BCL2/adenovirus E1B 19 kDa interacting protein 3 (BNIP3; **g** and **k**) and ANO6 (**h** and **l**). Arrows point at cilia

To determine the relevance of these findings for polycystic kidney disease in humans, we analyzed sections of human polycystic kidneys. In polycystic kidneys, increased apoptosis is a key feature of the cyst epithelium and highly correlates with the degree of cyst formation.^24^ ANO6 could be detected in human cyst-lining cells and—in line with the *in vitro* data—colocalized with the ciliary marker acetylated tubulin (Figures 7a–d). In addition, ANO6 was highly expressed in rounded apoptotic epithelial cells characterized by strong expression of the pro-apoptotic protein BNIP3 (Figures 7e–l), supporting the hypothesis of a functional role of ANO6 in apoptosis of cyst-lining epithelial cells.

## Discussion

The important function of the primary cilium is reflected by a strong compartmentalization. Thus, several proteins have been found to be exclusively or at least preferentially localized in the ciliary membrane or the ciliary lumen.^3^ In addition, primary cilia are characterized by distinct calcium concentrations compared with the cytosol regulated by calcium-permeable non-selective cation channels within the ciliary membrane.^25^ Here we show that ANO6, a calcium-activated chloride channel, is also preferentially localized in the cilium. In addition, we show that ANO6 is involved in cyst formation by mediating apoptosis-dependent cavitation, a prerequisite for proper lumen formation.

Ciliary localization of proteins depends on the recognition of a ciliary targeting sequence. The most common one is the VxP motif, which has been found in many ciliary transmembrane proteins including the polycystins.^26^ This sequence is also present in ANO6 and several other anoctamins, as shown in Supplementary Table 1. In line with our data showing a ciliary localization in canine and human tubular cells, the VxP motif of ANO6 is conserved among different species (Supplementary Table 2), emphasizing its functional relevance and suggesting that additional anoctamins may have a role in ciliary function.

In fact, ANO1, another member of the anoctamin chloride channel family, has recently also been shown to be localized in the cilium and to be involved in ciliogenesis.^27^ Unlike reported for ANO1, we found no evidence for a role of ANO6 in cilia formation, as cilia were well expressed in ANO6-knockdown cells. However, as our knockdown efficiency was about 80% we cannot rule out that complete knockout might affect ciliogenesis. In a previous report, we showed that ANO1 is involved in apical chloride secretion of cyst-forming renal cells, whereas knockdown of ANO6 had no effect on chloride secretion.^13^ These data are corroborated by findings in the current study, where cyst growth and cyst expansion were not affected by knockdown of ANO6. This might be explained by the fact that ANO6 needs a strong increase of intracellular Ca^2+^ (50–100 *μ*M) to mediate chloride conductance, which may only occur under pathological conditions such as apoptosis but not in viable cells upon administration of UTP.^20^ Thus ANO1 and ANO6, although sharing structural similarities, clearly have distinct functional properties in the context of cyst formation.

An intriguing question arising from our observations is, whether the pro-apoptotic function of ANO6 is related to its ciliary localization. Recently, we have shown that loss of Kif3a, a ciliary trafficking protein, also causes impaired lumen formation.^28^ However, this phenotype was not caused by loss of the cilium but due to misregulated microtubular cytoskeleton in the cell periphery.^28^ In addition, we found that in early stages of *in vitro* cyst development where apoptosis-dependent cavitation takes place, cilia are not yet present.^28^ Therefore, these findings indicate that the ciliary localization of ANO6 may not be a prerequisite for its effect on cyst lumen formation, but may be related to ANO6 located to the plasma membrane. It is tempting to speculate that ANO6 may have distinct functions depending on its localization within the cell.

Knockdown of ANO6 inhibited lumen formation of MDCK cysts owing to impaired apoptosis of luminal cells. Recent studies have highlighted the scramblase function of ANO6 during apoptosis in immune and blood cells which results in exposure of phosphatidylserines which then allows macrophages to recognize apoptotic cells.^20, 21, 22^ Beyond that, ANO6 has also been reported to mediate staurosporine- and cisplatin-induced programmed cell death in lymphocytes and Ehrlich-Lettre ascites cells, respectively.^29, 30^ Moreover, our findings suggest that ANO6 is also associated with epithelial apoptosis in human polycystic kidneys. Although at first sight counter-intuitive, previous studies have demonstrated that cyst-lining cells within polycystic kidneys show increased levels of apoptosis.^15^ In a simplified perspective, this has been attributed to the fact that increased cell proliferation which is a characteristic finding in cyst-lining cells also requires apoptosis as a complementary counterpart.^15^ According to this concept, lack of apoptosis would not allow regulated proliferation of cyst epithelial cells along the cyst walls. However, the underlying mechanism of ANO6-mediated apoptosis remains elusive at the moment. Interestingly, hypoxia has been identified as an additional mediator of apoptosis^31^ and both, ANO6 and BNIP3 are target genes of the hypoxia-inducible transcription factor HIF-1*α*.^32, 33, 34^ We have previously shown that HIF-1*α* is expressed in cyst-lining epithelial cells and is functionally involved in the progression of cyst growth through stimulation of calcium-dependent chloride secretion.^32, 35^ Thus, enhanced apoptosis mediated by increased expression of ANO6 could be an additional downstream mechanism of HIF-dependent cyst growth. Further *in vivo* analyses will be required to confirm a functional role of ANO6 in polycystic kidney disease.

## Materials and Methods

DMEM/Ham's F12 medium and modified MEM medium containing Earl's balanced salt solution was purchased from Biochrom AG (Berlin, Germany), DMEM medium and Hanks BSS from PAA Laboratories (Coelbe, Germany), insulin-transferrin-selenium supplement from Gibco (Karlsruhe, Germany), fetal calf serum (FCS) from PAN Biotech (Aidenbach, Germany), triiodothyronine from Fluka (Buchs, Switzerland), hydrocortisone from Sigma (Munich, Germany), epidermal growth factor from PeproTech (Hamburg, Germany).

### Cell culture

Human primary tubular epithelial cells (hPTECs) were isolated from renal cortical tissues collected from healthy parts of tumor nephrectomies as described previously.^17^ Isolation of human cells from healthy parts of tumor nephrectomies was approved by the local ethics committee. Cortex tissue was cut into 1 mm^3^ pieces and digested with collagenase type II (Gibco, Karlsruhe, Germany) and DNase I grade II (Roche Diagnostics, Mannheim, Germany) for 60 min. Next, cell suspension was sieved through 100 and 70 mm meshes. Cells were seeded in epithelial cell selective medium (DMEM/Ham's F12 medium containing 2 mM l-glutamine, 100 U/ml penicillin, 100 mg/ml streptomycin, insulin-transferrin-selenium supplement, 10 ng/ml epidermal growth factor, 36 ng/ml hydrocortisone and 4 pg/ml triiodothyronine) in the presence of 0.5% FCS. After 1–2 days, medium was replaced by FCS-free medium. MDCK cells were grown at 37 °C at 21% O_2_ and 5% CO_2_ and maintained in modified MEM containing Earl's balanced salt solution supplemented with 2 mmol/l l-glutamine, 10% heat-inactivated FCS, 50 IU/ml penicillin, and 50 mg/ml streptomycin. The human collecting duct HCD cell line was established from the normal part of a kidney removed for a localized adenocarcinoma as described previously.^16^ HCD cells were cultured at 37 °C in DMEM-Ham's F12 medium, supplemented with 5 *μ*g/ml transferrin, 50 nM sodium selenate, 2 mM glutamine, 5 × 10^−8^ M dexamethasone, 5 *μ*g/ml insulin, 2% FCS and 20 mM Hepes, pH 7.4. Polarized tubular epithelial cells were obtained by culturing cells for 6–8 days on permeable transwell inserts (Millicell, Millipore, Schwalbach, Germany) in the absence of FCS.

### Collection of human renal ADPKD tissue and patient characteristics

Kidney specimens of seven patients (six men, one woman; age, 55.6±9.3 years (mean±S.D.)) were obtained as described previously.^13^ Briefly, tissue was fixed immediately after nephrectomy in 3% paraformaldehyde (pH 7.4). Six patients were on hemodialysis at the time of nephrectomy, thus representing rather late stages of ADPKD. Collection and analysis of tissue samples were approved by the local ethics committee.

### shRNA and generation of ANO6-deficient cells

Primers complementary to two distinct regions of Canis familiaris ANO6 (accession number XP_852020.1) were cloned *Bgl*II and *Xho*I into the pSUPERIOR vector (Oligoengine, Seattle, WA). Correct cloning was verified by sequencing. As a negative control, pSUPERIOR containing a scrambled sequence was purchased from Oligoengine. MDCK cells were transfected with Fugene (Roche Diagnostics) according to the manufacturer's instructions. Colonies were picked after 2 weeks of treatment with G-418 (500 mg/ml; PAA Laboratories).

### Primer sequences used for shRNA directed against ANO6

The following primers were used for shANO6: 5′-GGATCCCCGCTTCCGTCATCAGCTTTATCTTCAAGAGAGATAAAGCTGATGACGGAAGCTTTTTCTCGAG-3′ and 5′-CTCGAGAAAAAGCTTCCGTCATCAGCTTTATCTCTCTTGAAGATAAAGCTGATGACGGAAGCGGGGATCC-3′ (sequence 1); and 5′-GGATCCCCGCCGCATTGTTTATTTCATCCTTCAAGAGAGGATGAAATAAACAATGCGGCTTTTTCTCGAG-3′ and 5′-CTCGAGAAAAAGCCGCATTGTTTATTTCATCCTCTCTTGAAGGATGAAATAAACAATGCGGCGGGGATCC-3′ (sequence 2).

### Cell proliferation assay

A total of 1000, 2500 and 5000 stable control-transfected MDCK (shControl) cells and MDCK cells stably deficient for ANO6 (shANO6#1 and shANO6#2) were seeded into 96-well plates. After 48 h, cells were fixed and stained with DAPI. Cell numbers of three individual experiments were counted by the use of ImageJ (V.1.45, U.S. National Institutes of Health, Bethesda, MD, USA).

### Annexin V binding assay

Stable control-transfected MDCK (shControl) cells and MDCK cells stably deficient for ANO6 (shANO6#1 and shANO6#2) were grown on glass cover slips and treated with 0, 0.1 or 1 mM ATP. After 15 min, cells were incubated with annexin V-FITC (BD Pharmingen, Heidelberg, Germany) for 15 min at 20 °C. Cells were subsequently analyzed by the use of a BZ-9000 microscope (Keyence, Osaka, Japan) and ImageJ (V.1.45, U.S. National Institutes of Health).

### MDCK cyst model

*In vitro* cyst assays were performed as described previously.^36, 37^ In brief, MDCK cells were resuspended as a single-cell suspension in type I collagen and filled into 24-well plates (three to six wells per condition). Forskolin (10 *μ*M; Sigma-Aldrich) was added to the medium when indicated in the figures at day 0, and medium was changed every 2 days. After 5 days, two random visual fields per well were photographed with an Olympus CK40 microscope ( × 40 magnification; Olympus Lifes Science Research GmbH, Munich, Germany) and a Leica DC200 camera (Leica Microsystems, Wetzlar, Germany). Cyst diameters as well as the circumferences of the lumina and the cysts (~80–360 cysts per condition and single experimental procedure) were measured with ImageJ (V. 1.45, U.S. National Institutes of Health) and the use of a Wacom Tablet device. Cyst volume was then estimated using the formula for the volume of a sphere, 4/3πr^3^.

### Ussing chamber experiments

MDCK cells were grown as polarized monolayers on permeable supports (Millipore) for 9 days. Cells then were mounted into a perfused micro Ussing chamber and the luminal and basolateral surfaces of the epithelium were perfused continuously with ringer solution (in mM: NaCl (145), KH_2_PO_4_ (0.4), K_2_HPO_4_ (1.6), glucose (5), MgCl_2_ (1) Ca-gluconate (1.3)) at a rate of 6 ml/min (chamber volume 2 ml). In addition, 10 *μ*M UTP were added on the apical side or 100 *μ*M 3-isobutyl-1-methylxanthine and 2 *μ*M Forskolin (I/F) were added on the basolateral side as indicated in the figure. All the experiments were carried out at 37 °C under open-circuit conditions. Transepithelial resistance (Rte) was determined by applying short (1 s) current pulses (Δ*I*=0.5 μA) and the corresponding changes in transepithelial voltage (Vte) were recorded continuously. Values for Vte were referred to the serosal side of the epithelium. Rte was calculated according to Ohm's law (Rte=ΔVte/Δ*I*). The equivalent short-circuit current (Isc) was calculated according to Ohm's law from Vte and Rte (Isc=Vte/Rte).

### ANO6 antibodies

Affinity-purified polyclonal antisera against ANO6 were produced in rabbits immunized with three different peptides corresponding to mouse or human ANO6 (listed in Supplementary Table 4) coupled to keyhole limpet hemocyanin (Davids Biotechnologie, Regensburg, Germany). Antibodies 1–3 were used for immunocytochemistry of ANO6 in MDCK cells (Supplementary Figure 1). Staining was almost completely abolished, when ANO6 was deleted by stable knockdown of ANO6 in MDCK cells (Figure 2). Antibodies 2 and 3 detected comparable signals for ANO6 in human cells (Supplementary Figure 2). Cross-reaction between the primary antibody directed against acetylated tubulin or the secondary antibody used to detect acetylated tubulin with the secondary antibody used to detect ANO6 was excluded in all cells used in the experiments (Supplementary Figure 4).

### Immunofluorescence

Cells kept on permeable inserts as well as MDCK cysts were rinsed in PBS supplemented with 0.9 mM calcium chloride and 0.49 mM magnesium chloride (PBS+). Paraformaldehyde (4%) was added to fix the cells and cysts for 1 h at RT. Glycine (200 mM) in PBS+ was added for another hour to quench the excess aldehyde. Blobs of collagen gel were put into biopsy bags and paraffinized. MDCK cysts were stained for DNA strand breaks (TUNEL; *in situ* cell death detection kit; Roche) activated Caspase 3 (1 : 100; rabbit; Epitomics) and F-actin conjugated to AlexaFluor 488 (1 : 100; Invitrogen, Darmstadt, Germany). If not stated differently in the figure legend, cells were stained for ANO6 by the use of ANO6_ab1 (1 : 200; rabbit; Supplementary Table 4), human kidney sections were stained with ANO6_ab2 (1 : 300; rabbit; Supplementary Table 4). Both, cells and sections were stained for acetylated tubulin (1 : 300; mouse; Sigma-Aldrich). In addition, cells were stained for E-cadherin (1 : 200; rabbit; Santa Cruz, Heidelberg, Germany) and sections were stained for BNIP3 (1 : 400; mouse; Abcam, Cambridge, UK). Binding of the primary antibody was visualized by incubation with secondary anti-rabbit antibody conjugated with AlexaFluor 555 or 488 or anti-mouse antibody AlexaFluor 488 (each 1 : 500, Molecular Probes, Darmstadt, Germany, Invitrogen). Immunofluorescent signals were captured with a BZ-9000 microscope (Keyence, Osaka, Japan) and the background correction algorithm in ImageJ (V.1.45, U.S. National Institutes of Health) was applied. Colocalization was visualized in white by the use of ImageJ (V.1.45, U.S. National Institutes of Health) and the colocalization finder algorithm (http://rsb.info.nih.gov/ij/plugins/colocalization-finder.html) by the authors Christophe Laummonerie, Jerome Mutterer, Institut de Biologie Moleculaire des Plantes, Strasbourg, France.

### Quantification of ANO6 intensities in the MDCK cyst epithelium

In order to quantify the fluorescence intensities of ANO6 within the epithelium of MDCK cysts *n*=20 control cysts and *n*=26 forskolin-treated cysts originating from three independent experiments were stained for ANO6 and photographed by the use of a confocal microscope TCS SP5 II (Leica Microsystems, Wetzlar, Germany). Within each cyst, four random regions of interest were selected capturing the fluorescence profile from the basal to the apical membrane using ImageJ (V.1.45, U.S. National Institutes of Health) with the investigator being blinded to the experimental condition. Mean basal, central and apical fluorescence was determined and averaged for every single cyst.

### Real-time PCR

Total RNA (1 *μ*g) isolated from MDCK cells were reverse-transcribed using random primer and M-MLV Reverse Transcriptase RNase H Minus (Promega, Mannheim, Germany). Real-time reverse transcriptase-polymerase chain reaction was performed in a plate reader Light Cycler 480 by using a Sybrgreen I PCR Kit (Roche Applied Science, Mannheim, Germany) and specific primer (Supplement Table 3).

### Statistical analysis

Data are expressed as mean±S.E.M. The differences among groups were analyzed using one-way ANOVA, followed by a Bonferroni test for multiple comparisons. An unpaired *t*-test was applied to compare the differences between two groups, a paired *t*-test was used for matched observations. Wilcoxon signed-rank test for columns statistics was used for relative values. *P*<0.05 was considered statistically significant and marked with an asterisk (*) in the figures.

## Abbreviations

ANO: anoctamin
MDCK: Madin-Darby Canine Kidney
TMEM: transmembrane protein
sh: short hairpin
Ctrl: control
HCD: human collecting duct
ATP: adenosine triphosphate
IBMX: 3-isobutyl-1-methylxanthine
TUNEL: TdT-mediated dUTP-biotin nick end labeling
BNIP3: BCL2/adenovirus E1B 19 kDa interacting protein 3
UTP: uridine triphosphate
Kif3a: kinesin family member 3A
HIF-1*α*: hypoxia-inducible factor 1, alpha subunit
DMEM: Dulbecco's modified Eagle's medium
BSS: balanced salt solution
FCS: fetal calf serum
hPTECs: human primary tubular epithelial cells
ADPKD: autosomal dominant polycystic kidney disease
FITC: fluorescein isothiocyanate
Rte: transepithelial resistance
Vte: transepithelial voltage
Isc: short-circuit current
PBS: phosphate-buffered saline
PBS+: PBS supplemented with 0.9 mM calcium chloride and 0.49 mM magnesium chloride
S.E.M.: standard error of the mean
Fsk: forskolin
DIC: digital interference contrast
