# Supplementary figures and images for: Anoctamin 6 is localized in the primary cilium of renal tubular cells and is involved in apoptosis-dependent cyst lumen formation
Source: Cell Death Dis. 2015 Oct 8;6(10):e1899–. doi: 10.1038/cddis.2015.273 (PMC4632301; doi:10.1038/cddis.2015.273)

Supplemental Figure 1

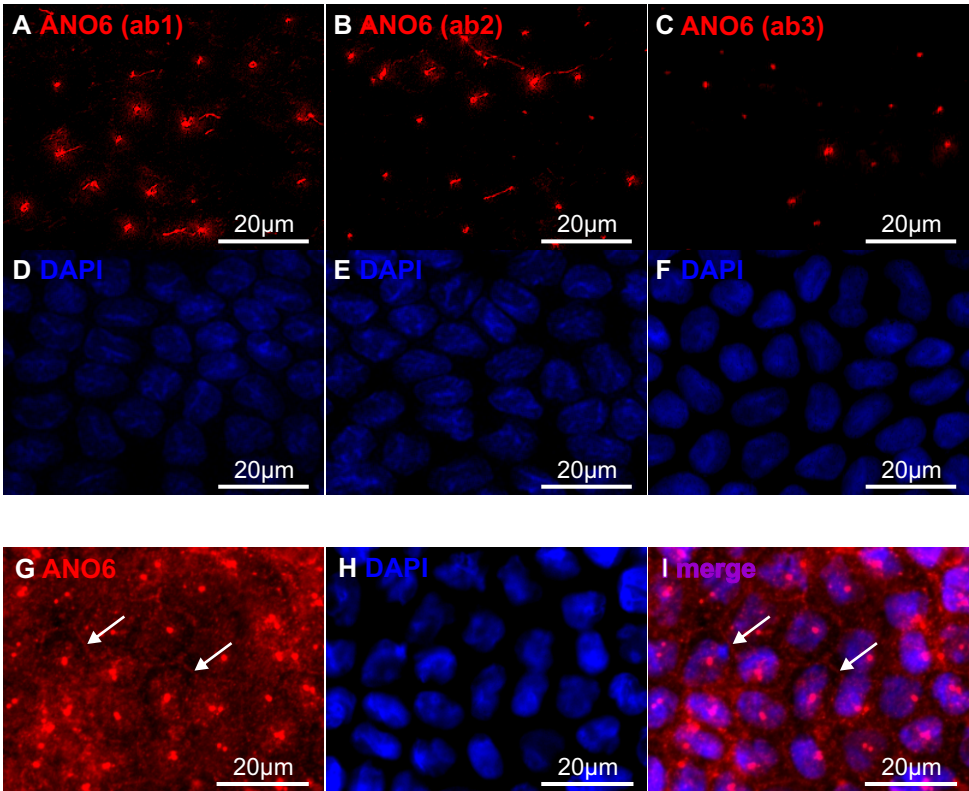

## Supplemental Figure 2

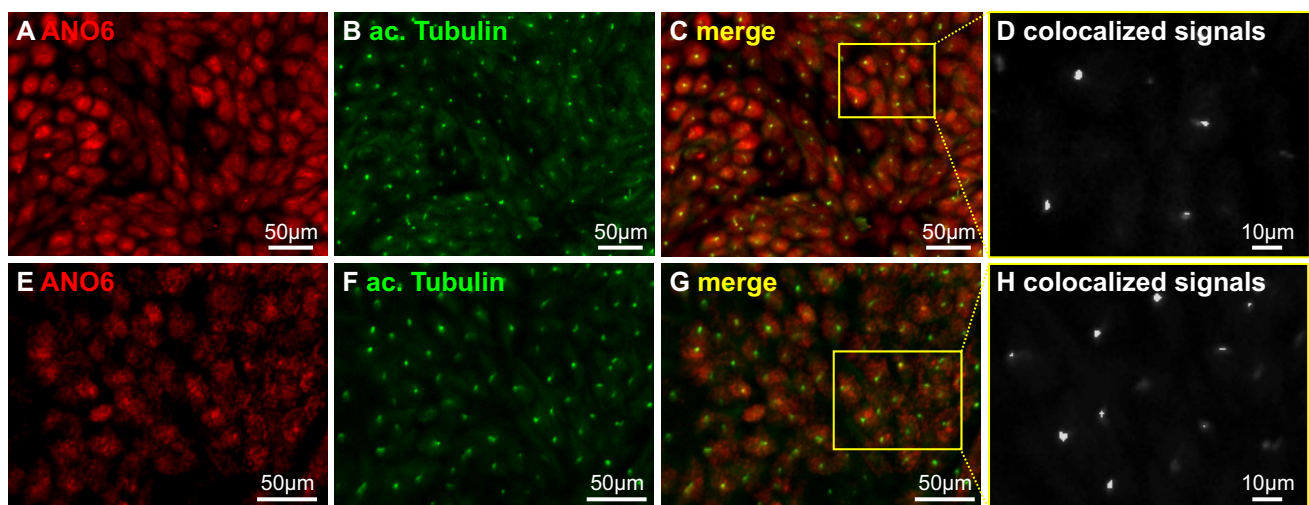

# Supplemental Figure 3

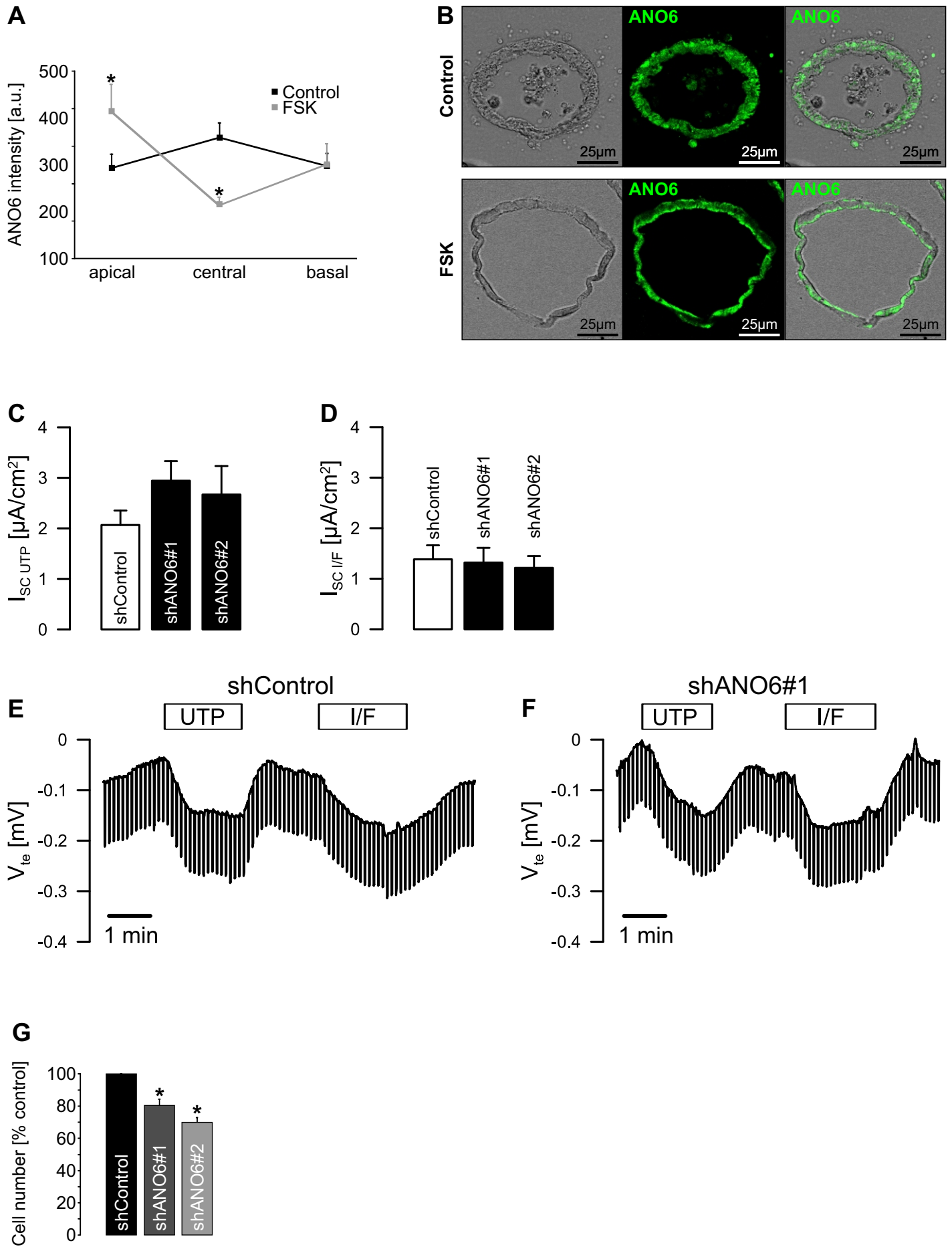

Supplemental Figure 4

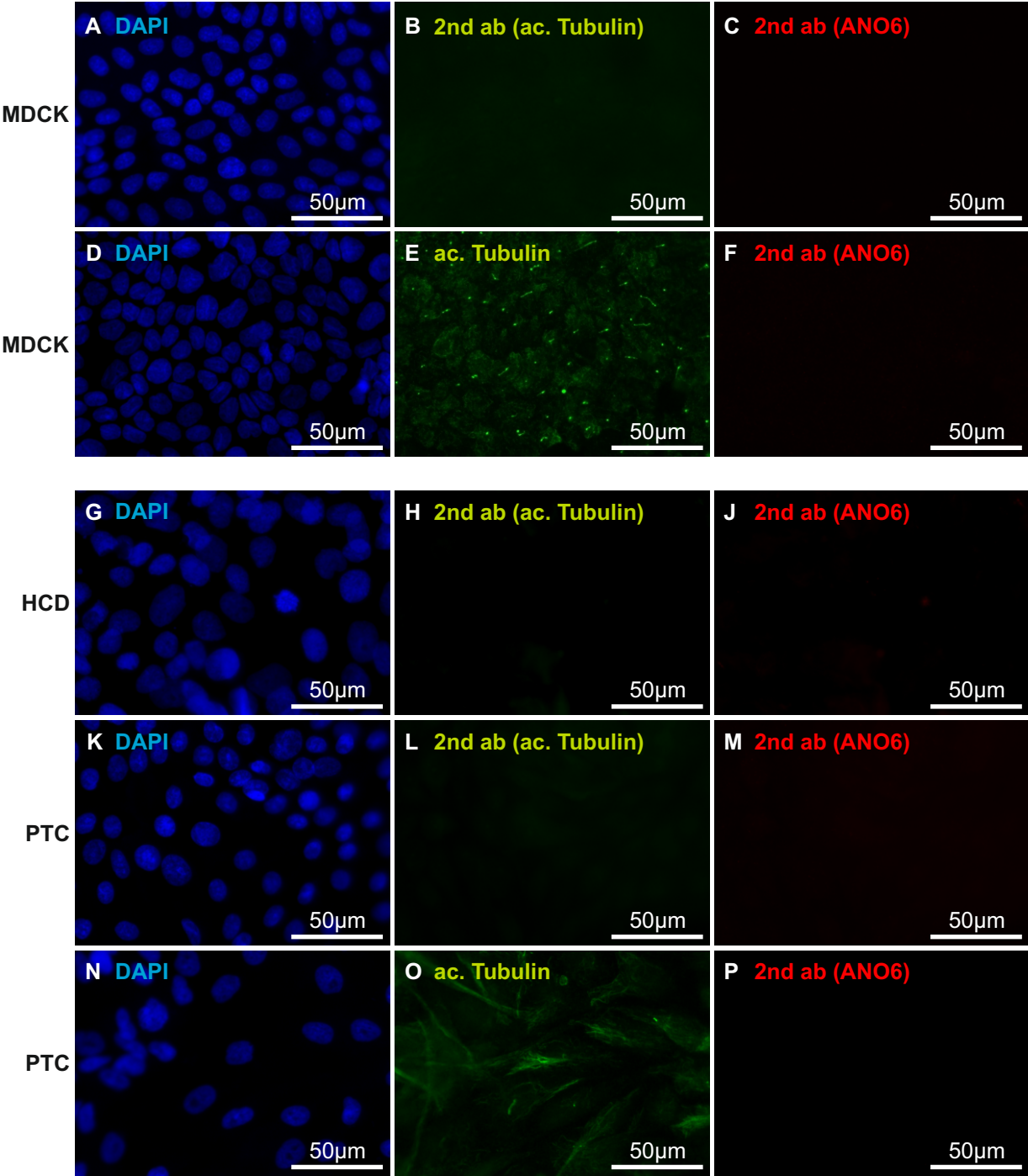

Supplement: Supplementary Figures [file cddis2015273x1.pdf]
