# Supplementary material for: Anoctamin 6 is localized in the primary cilium of renal tubular cells and is involved in apoptosis-dependent cyst lumen formation
Source: Cell Death Dis. 2015 Oct 8;6(10):e1899–. doi: 10.1038/cddis.2015.273 (PMC4632301; doi:10.1038/cddis.2015.273)
Supplement: Supplementary Information [file cddis2015273x2.doc]

**Supplement**

**S. Table 1: VxP motif in human Anoctamins**

| **Anoctamin** | **VxP motif** |
| --- | --- |
| ANO1 | aa 43-45  aa 911-913 |
| ANO2 | - |
| ANO3 | aa 527-529  aa 854-856 |
| ANO4 | - |
| ANO5 | - |
| ANO6 | aa 761-763 |
| ANO7 | aa 71-73  aa 219-221  aa 223-225 |
| ANO8 | aa 208-210  aa 260-262 |
| ANO9 | aa 11-13 |
| ANO10 | aa 170-172  aa 514-516  aa 583-585 |

S. Table 2: ANO6 displays conserved VxP-motif

| Homo sapiens | aa 761-763 |
| --- | --- |
| Canis | aa 779-781 |
| Rattus norvegicus | aa 774-776 |
| Mus musculus | aa 34-36  aa 906-908 |

**S. Table 3: Realtime Primer**

| ANO6 | sense: 5´- CAACCTATTGGCTGGGAAAA  antisense: 5´- TCAGCTGTGTCGTCAGTTCA |
| --- | --- |
| 18s | sense: 5´- TGATTAAGTCCCTGCCCTTTGTA  antisense: 5´- CGATCCGAGGGCCTCACTA |
| Hprt1 | sense: 5´- CGGCTTGCTCGAGATGTGAT  antisense: 5´- GAGCACACAGAGGGCTACGAT |

**S. Table 4: Anoctamin 6 Antibodies**

| **Antibody** | **Epitope** | **Localization of the epitope** |
| --- | --- | --- |
| ANO6_ab1 | mouse: KREKYLTQKLLHESHLKDLTK | 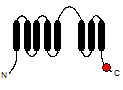  AA 869-889 |
| ANO6_ab2 | human: REKYLTQKLLHENHLKDMTK | 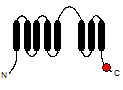  AA 869-888 |
| ANO6_ab3 | human: KVADFKNKSKGNPYSDLGNHT | 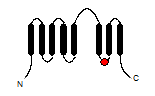  AA 784-804 |

All three antibodies were used for staining of ANO6 in MDCK cells, and antibody 2 and -3 were used for staining of ANO6 in HCD cells and primary human tubule cells, each providing similar results.

**Supplemental Figure 1. Anoctamin staining can be reproduced by three different antibodies. Anoctamin 6 is localized in the plasma membrane of MDCK cells. A-F** MDCK cells grown on permeable supports were stained for ANO6 by the use of antibody ANO6_ab1 (ab1, A), ANO6_ab2 (ab2, B) and ANO6_ab3 (ab3, C) listed in supplemental table 4. **D-F** Nucleus staining (DAPI) confirmed high confluence of the analysed cells described in A-C. **G-I** MDCK cells grown on permeable supports stained for ANO6 but longer exposed than in Figure 1 or A-C showing positive plasma membrane signal (arrows). **H** shows nuclei from cells in A. **I** shows merged photo of (A) and (B).

**Supplemental Figure 2. ANO6 localizes in the primary cilium of human primary tubule cells.** Primary human renal tubule cells were stained for ANO6 with antibody ANO6_ab2 (**A**) and antibody ANO6_ab3 (**E**), and acetylated tubulin (**B,F**). **C** and **G** show merged photos of (A) and (B), and (E) and (F), respectively. **D** and **H** show calculated colocalization highlighted in white of the magnified sections marked in (C) and (G), respectively.

**Supplemental Figure 3. Forskolin leads to translocation of ANO6 to the apical cell membrane of MDCK cells (A,B) but is not involved in chloride secretion (C-F). Knockdown of ANO6 results in decreased cell proliferation of MDCK cells (G). A** MDCK cells were grown within a collagen I matrix in the presence and absence of 10µM forskolin (FSK) to form cysts for 5 days.Thereafter, cysts were stained for ANO6 and fluorescence intensities from 4 regions of 20-26 cysts per condition out of 3 individual experiments were analyzed at the luminal membrane, the center, and the basal membrane of the cyst-epithelial cells. **B** Representative photos of MDCK cysts at day 5 stained for ANO6 (green). **C** Summary of the mean short circuit currents (Isc) of MDCK cells stably deficient for ANO6 (shANO6#1 and shANO6#2) compared with control-transfected MDCK cells (shControl) upon incubation with 10 µM UTP applied on the apical side. **D** Mean Isc of cells described in C upon incubation with 100 µM 3-isobutyl-1-methylxanthine and 2 µM Forskolin (I/F) applied on the basolateral side. C,D: n=6 individual experiments. **E,F** Representative original recordings of open-circuit Ussing chamber experiments using control-transfected MDCK cells (E), and ANO6-deficient cells (F). **G** Identical cell numbers of stably control-transfected MDCK cells (shControl) and MDCK cells stably deficient for ANO6 (shANO6#1 and shANO6#2) were seeded in 96-well plates in three individual experiments. 48 h later, cell numbers were counted and referred to shControl (set = 100%).

**Supplemental Figure 4. ANO6 staining cannot be referred to cross-reactivity of the primary or secondary antibody used to stain for acetylated tubulin with the secondary antibodies used to detect ANO6. A-F** MDCK cells, **G-J** HCD cells, and **K-P** human primary tubular cells (PTC) were stained for nuclei (DAPI, blue) and incubated with the secondary anti-mouse antibody AlexaFluor® 488 used to detect acetylated tubulin (2nd ab (ac. Tubulin); green) in the presence or absence of the primary antibody directed against acetylated tubulin (ac. Tubulin). In addition, all sections were incubated with the secondary anti-rabbit antibody conjugated with AlexaFluor® 555 (2nd ab (ANO6); red) used to detect binding of the primary anti-ANO6 antibody.
